# Dynamic expression of IGFBP3 modulate dual actions of mineralization micro-environment during tooth development via Wnt/beta-catenin signaling pathway

**DOI:** 10.1186/s13062-023-00391-9

**Published:** 2023-06-26

**Authors:** MengDan Zhang, Junming Zheng, Siyuan Wu, Hailing Chen, Lusai Xiang

**Affiliations:** 1 Guanghua School of Stomatology, Hospital of Stomatology, Guangdong Provincial Key Laboratory of Stomatology, Sun Yat-Sen University, No. 56 Lingyuan West Road, Guangzhou, 510055 Guangdong China; 2 Foshan Stomatological Hospital, School of Stomatology and Medicine, Foshan University, No. 5, Hebin Road, Chancheng District, Foshan, 528000 Guangdong China

**Keywords:** Insulin-like growth factor binding protein 3, Mineralization micro-environment, Tooth development, Wnt/beta-catenin, DKK1

## Abstract

**Background:**

Tooth development, as one of the major mineralized tissues in the body, require fine-tuning of mineralization micro-environment. The interaction between dental epithelium and mesenchyme plays a decisive role in this process. With epithelium–mesenchyme dissociation study, we found interesting expression pattern of insulin-like growth factor binding protein 3 (IGFBP3) in response to disruption of dental epithelium–mesenchyme interaction. Its action and related mechanisms as regulator of mineralization micro-environment during tooth development are investigated.

**Results:**

Expressions of osteogenic markers at early stage of tooth development are significantly lower than those at later stage. BMP2 treatment further confirmed a high mineralization micro-environment is disruptive at early stage, but beneficial at later stage of tooth development. In contrast, IGFBP3's expression increased gradually from E14.5, peaked at P5, and decreased afterwards, demonstrating an inverse correlation with osteogenic markers. RNA-Seq and Co-immunoprecipitation showed that IGFBP3 regulates the Wnt/beta-catenin signaling pathway activity by enhancing DKK1 expression and direct protein–protein interaction. The suppression of the mineralization microenvironment effectuated by IGFBP3 could be reversed by the DKK1 inhibitor WAY-262611, further demonstrating that IGFBP3 exerted its influence via DKK1.

**Conclusion:**

A deeper understanding of tooth development mechanisms is essential for tooth regeneration, which have great implications for dental care. The current study demonstrated that the IGFBP3 expression is regulated in accordance with the needs of the mineralization microenvironment during tooth development, and IGFBP3 exerts its modulating action on osteogenic/odontogenic differentiation of hDPSCs by DKK1-Wnt/ beta-catenin axis.

**Supplementary Information:**

The online version contains supplementary material available at 10.1186/s13062-023-00391-9.

## Background

As one of the major mineralized tissues in the body, tooth development requires a fine tuning of mineralization micro-environment. Its detailed mechanisms, especially how it is regulated differentially during various stages and locations during tooth development, are still unclear. Epithelial mesenchymal interactions (EMI) play an essential role in tooth morphogenesis, culminating in a process of programmed, sequential, and reciprocal communications that participate in all aspect of tooth development, including regulation of mineralization micro-environment [1]. Epithelium mesenchyme dissociation and recombination study provide an interesting perspective to study epithelial mesenchymal interactions [2], Our previous study showed that the removal of dental epithelium disrupted tooth morphogenesis, and changed the expression pattern of the dental mesenchyme, creating a mineralization inducing microenvironment [3]. The spatiotemporal expression pattern of these bioactive factors is not only important for the understanding of tooth development but also holds the key to tooth regeneration. How these factors influence the mineralization microenvironment in the dental mesenchyme are worth further investigation.

Our tooth germ dissociation study, combined with protein microarray assay found that removal of dental epithelium altered insulin-like growth factor binding protein 3 (IGFBP3) level prominently. IGFBP3 is a member of the IGFBP gene family. It can bind with IGF-I, forming the IGF-IGFBP complex, which is crucial for regulation of the IGF-I turnover, transport, and distribution [4]. IGF-I is known to promote differentiation of human dental papilla stem cells [5, 6]. IGFBP3 regulates the transition from the proliferative to the differentiation stage in dental papilla cells via interaction with IGF-I [7]. IGFBP3 also plays IGF-independent roles, which are usually mediated through binding to the matrix, cell-surface, cytoplasmic, nuclear, and mitochondrial molecules [8]. However, only a limited number of studies address the expression and role of IGFBP3 in tooth development.

In the present study, we confirmed IGFBP3 as the protein mainly secreted by the dental mesenchyme under the stimulation of dental epithelium. The spatiotemporal expression pattern of IGFBP3, as well as various odontogenic and osteogenic markers were evaluated. The influence of IGFBP3 on the osteogenic and odontogenic differentiation of hDPSCs was assessed with both in vitro and in vivo experiments. Finally, the mechanisms underlying IGFBP3’s action were investigated.

## Results

### Spatiotemporal expression profiles of IGFBP3 and osteogenic markers during tooth development

**Fig. 1:**
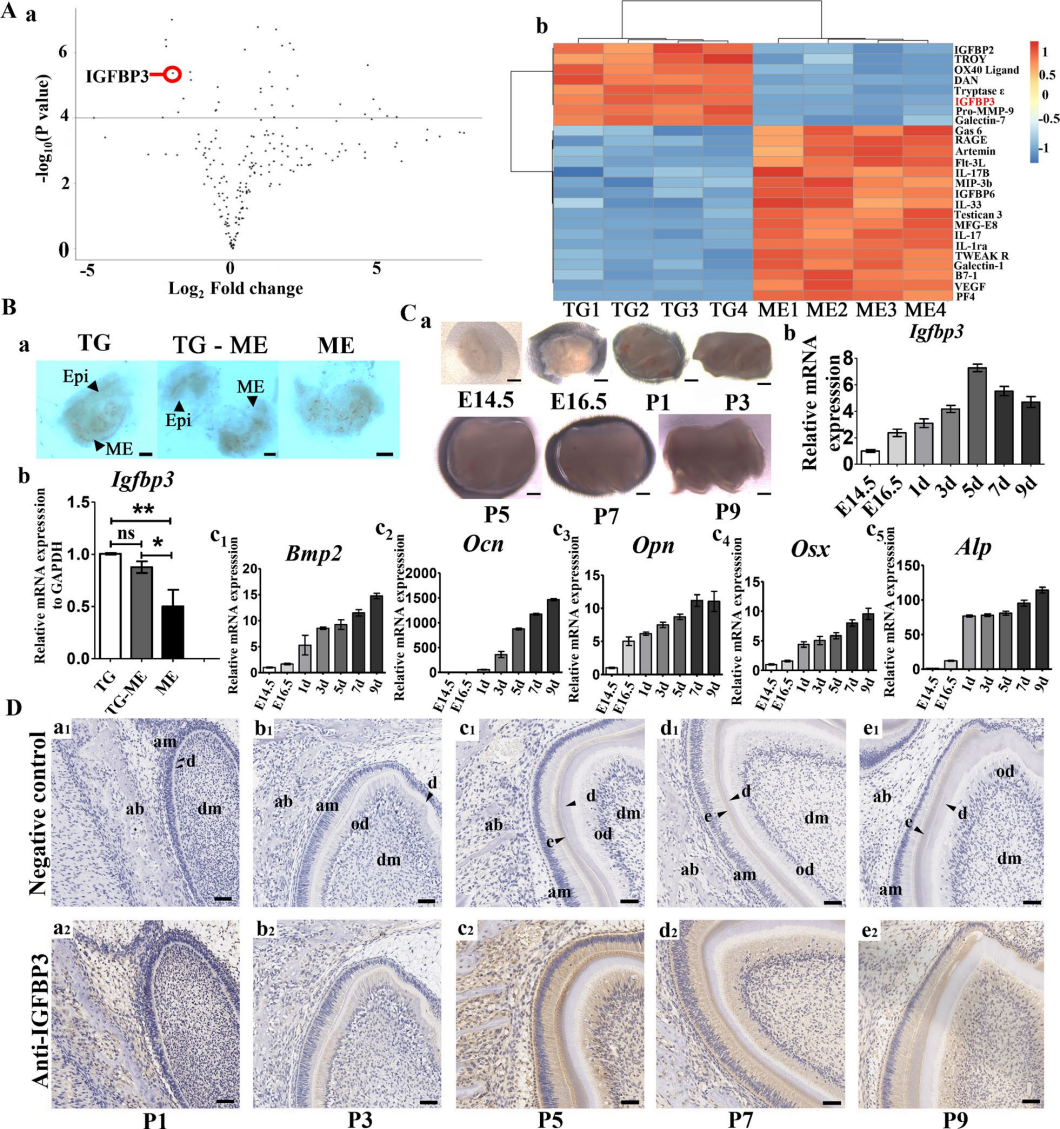
The spatiotemporal expression pattern of IGFBP3. **A** Volcano plot (a, ME vs. TG) and heatmap (b) demonstrating extracellular protein level difference introduced by dental epithelium–mesenchyme dissociation. **B** Isolation of tooth germ tissue from E14.5 mouse embryo mandibles and subsequent dissociation study (a). RT-qPCR analysis compared Igfbp3 levels in each group (b). **C** Tooth germs of first molars were isolated from E14.5-P9 mandibles (a). The mRNA levels of *Igfbp3* (b) and osteogenic markers *Bmp2* (c_1_), *Ocn* (c_2_), *Opn* (c_3_), *Osx* (c_4_) and *Alp* (c_5_) in tooth germs from E14.5 to P9 were detected by RT-qPCR. **D** Immunohistochemical analyses were performed to evaluate the expression of IGFBP3 at different stages of tooth development. *ME* dental mesenchyme alone, *TG* whole tooth germ, *TG-ME* dental mesenchyme isolated after organ culture; *Bmp2*, bone morphogenetic protein 2; *Ocn*, osteocalcin; *Opn*, osteopontin; *Osx*, osterix; *Alp*, alkaline phosphatase; ab, alveolar bone; am, ameloblasts; d, dentin; e, enamel; dm, dental mesenchyme; od, odontoblast. n = 3, *p < 0.05, **p < 0.01, ns: no significance. Scale bars: 750 μm (**C**-a), 50 μm (**D**)

Protein microarray analysis compares secreted proteins levels between the whole tooth germ (TG) and dental mesenchyme alone (ME). Results show that removal of the dental epithelium changes the expression of various secreted proteins. Among them, IGFBP3 stands out, as its level is significantly (Fig. 1A a) and consistently (Fig. 1A b) decreased in the ME group.

The spatial pattern for IGFBP3 expression was validated with a RT-qPCR study. Tooth germs isolated at E14.5 were divided into three groups (Fig. 1B a): the whole tooth germ group (TG), dental mesenchyme alone group (ME), and dental mesenchyme isolated after culture group (TG—ME). The tissue in each group was cultured for 7 days before the RT-qPCR assay, and for the TG-ME group, only isolated dental mesenchyme tissue was used for the RT-qPCR assay. The IGFBP3 level was significantly lower in the ME group than in the TG group, which is consistent with the protein microarray result. Interestingly, the IGFBP3 level in the mesenchymal part of whole tooth germ (TG-ME) was similar to that of whole tooth germ (Fig. 1B b). These results indicate that in the tooth germ, IGFBP3 is predominantly expressed in the mesenchymal region, and removal of dental epithelium at E14.5 leads to its decreased expression.

First mandibular molar tooth germs dissected from E14.5, E16.5 Chinese Kunming mice embryos and P1–P9 Chinese Kunming mice (Fig. 1C a) were evaluated for Igfbp3 and osteogenic markers with RT-qPCR. The IGFBP3 level increased gradually from E14.5, peaked at P5, and decreased afterwards (Fig. 1C b). For osteogenic markers, the expression level increased continuously from E14.5 to P9 (Fig. 1C c1–c5), forming an inverse correlation with the IGFBP3 level at a later stage of development (P5–P9). Immunohistochemical analyses were performed to further evaluate the expression of IGFBP3 at different stages of tooth development. IGFBP3 expression was found in both the epithelium and dental mesenchyme. It increased gradually from the early stage of tooth development and began to decrease on post‑partum Day 5 (Fig. 1D), which is consistent with our findings by RT-qPCR.

### Influence of BMP2 on development of tooth germs at different stages of tooth morphogenesis

**Fig. 2:**
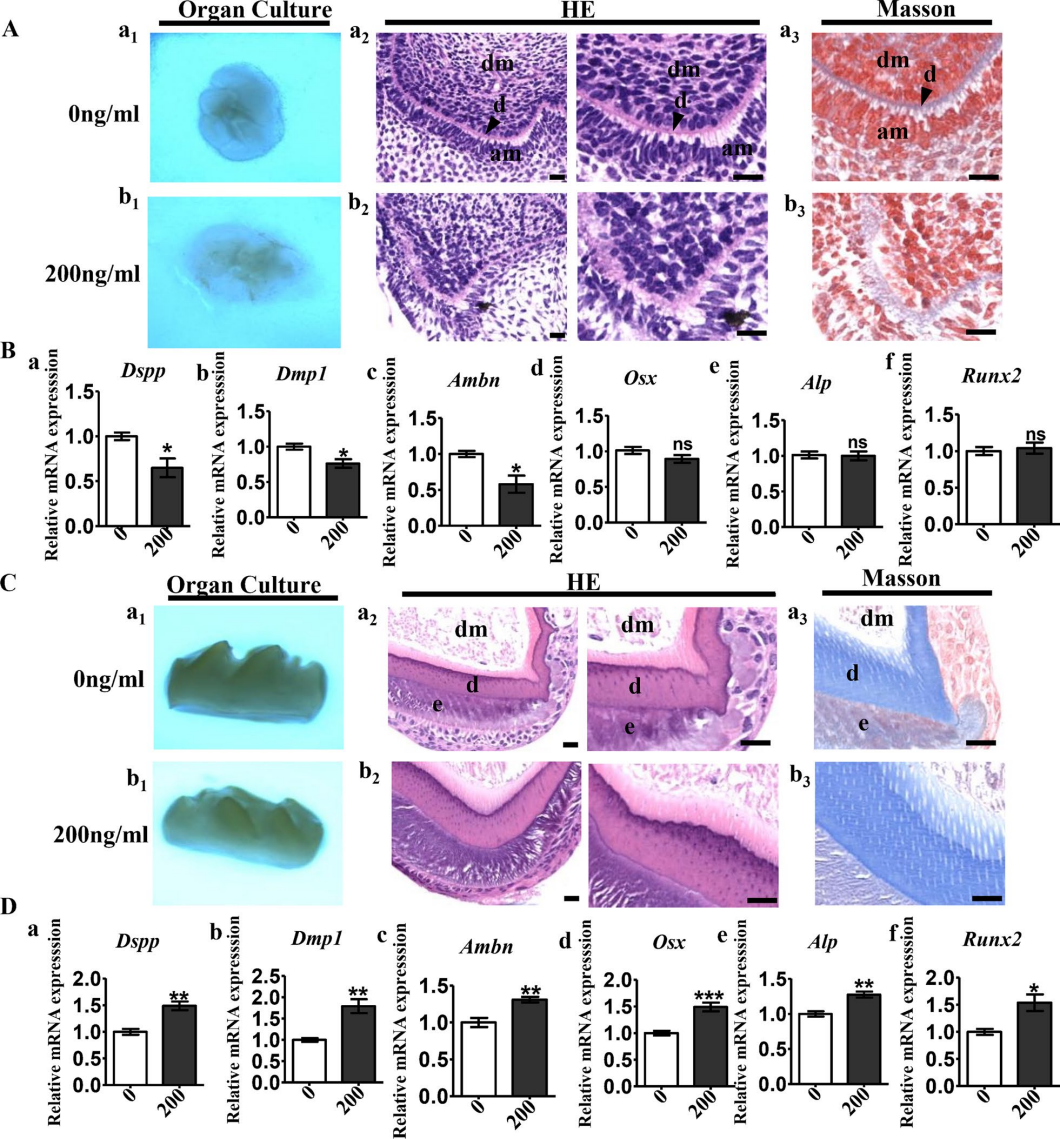
Mineralization microenvironment’s influence on tooth germ isolated at E14.5 and P7. **A** Tooth germs of first molars isolated from E14.5 mandibles were incubated with (200 ng/ml, b_1_) or without (0 ng/ml, a_1_) BMP2 for 7 days. HE staining of tissue sections demonstrated structure disruption with loss of polarity and continuity after stimulation with 200 ng/ml of BMP2 (b_2_) compared with control (a_2_). Masson’s trichrome staining high-lighted the disruption to the dentin structure in 200 ng/ml BMP2 treated group (b_3_) compared with control group (a_3_). **B** RT-qPCR comparing tooth development related markers, including *Dspp*(a), *Dmp1*(b), *Ambn*(c), and osteogenic markers including *Osx*(d), *Alp*(e), and *Runx2* (f) between 200 ng/ml BMP2 treated E14.5 tooth germ and control group. **C** Tooth germs of first molars isolated from P7 mandibles were incubated with (200 ng/ml, b_1_) or without (0 ng/ml, a_1_) BMP2 for 7 days. HE staining and Masson's trichrome staining indicated increased dentin formation in 200 ng/ml BMP2 treated group (b2 and b3) compared with control group (a2 and a3). **D** RT-qPCR comparing tooth development related markers and osteogenic markers including between 200 ng/ml BMP2 treated P7 tooth germ and control group. *Dspp*, dentin sialophosphoprotein; *Dmp1*, dentin matrix acidic phosphoprotein 1; *Ambn,* ameloblastin; *Osx*, osterix; *Alp*, alkaline phosphatase; *Runx2*, Runt-related transcription factor 2. n = 5, *p < 0.05, **p < 0.01, ***p < 0.001. *ns* no significance. Scale bars: 200 μm (**A**, **C**)

Recombinant mouse BMP2 protein (200 ng/mL) was added to the culture medium of the first molar tooth germ from E14.5 and P7 Chinese Kunming Mice to simulate a high-mineralization micro-environment. For the E14.5 tooth germ, compared with the control group (Fig. 2A a1, a2), 200 ng/mL BMP2 caused considerable structure disruption with loss of polarity and continuity (Fig. 2A b1, b2). Masson’s trichrome staining further high-lighted the disruption to the dentin structure in 200 ng/mL BMP2 treated tooth germ (Fig. 2A b3), compared with the control group (Fig. 2A a3). The RT-qPCR result showed that for the E14.5 tooth germ, 200 ng/mL BMP2 treatment suppressed tooth development related markers (Fig. 2B a–c), including dentin sialophosphoprotein (Dspp), dentin matrix acidic phosphoprotein 1(Dmp1), and ameloblastin (Ambn). However, for osteogenic markers, including Osx, Alp, and Runx2, no significant difference was observed (Fig. 2B d–f).

For postnatal 7d tooth germs, comparing with control group (Fig. 2C a1–a3), both HE staining and Masson’s trichrome staining indicated increased dentin formation (Fig. 2C b1–b3). The RT-qPCR result showed that both odontogenic markers (Dspp, Dmp1, Ambn, Fig. 2D a–c) and osteogenic markers (Osx, Alp, Runx2, Fig. 2D d–f) were up-regulated after 200 ng/mL BMP2 treatment.

### Increased IGFBP3 levels inhibits odontogenic and osteogenic differentiation of human dental papilla stem cells and dental mesenchyme.

**Fig. 3:**
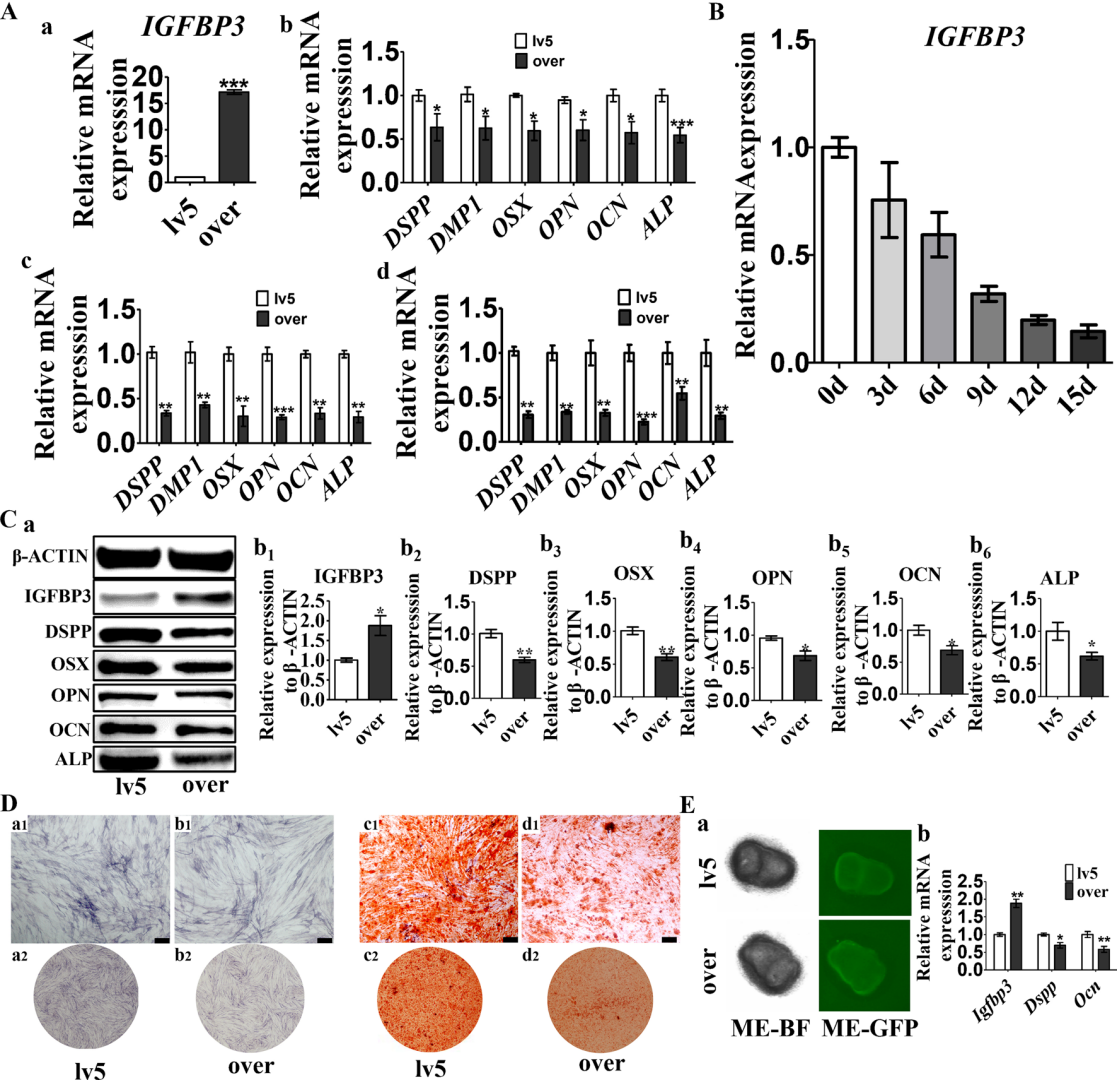
Effects of over-expressed IGFBP3 on odontogenic and osteogenic differentiation of human dental papilla stem cells and dental mesenchyme. **A** *IGFBP3* expression in lv5 hDPSCs versus IGFBP3-over hDPSCs after lentivirus transfection (a); Relative expression of *DSPP*, *DMP1*, *OSX*, *OPN*, *OCN* and *ALP* in lv5 hDPSCs versus IGFBP3-over hDPSCs after 0 days (b), 4 days (c) and 7 days (d) osteogenic induction. **B** The *IGFBP3* expression response to osteogenic induction was investigated by RT-qPCR using hDPSCs. **C** The protein levels of IGFBP3, odontogenic and osteogenic markers in lv5 hDPSCs and IGFBP3-over hDPSCs were detected by Western blot analysis (a). Quantitative analysis of the relative protein expression in IGFBP3 (b_1_), DSPP (b_2_), OSX (b_3_), OPN (b_4_), OCN (b_5_) and ALP (b_6_). **D** Alizarin red staining of lv5 hDPSCs and IGFBP3-over hDPSCs with 14 days osteogenic induction; Alkaline phosphatase staining of lv5 hDPSCs and IGFBP3-over hDPSCs treated with osteogenic induction medium for 7 days. Microscopic views: a_1_–d_1_, scale bar 200 μm; gross views: a_2_–d_2_. **E** Microscopic views of dental mesenchymal tissue from the P1 tooth germ with lentivirus transfection (a). Relative expression of *Igfbp3*, *Dspp*, *Ocn* in lv5 dental mesenchyme and IGFBP3-over dental mesenchyme were detected by RT-qPCR (b). lv5, hDPSCs or dental mesenchyme with empty pGLV5 vector control; over, hDPSCs or dental mesenchyme with IGFBP3 overexpressing vector. n = 3, *p < 0.05, **p < 0.01, ***p < 0.001

The IGFBP3 expression in hDPSCs was manipulated with lentivirus and confirmed with RT-qPCR assays (Fig. 3A a), generating hDPSCs with over-expressed IGFBP3 (denoted as IGFBP3-over hDPSCs) and normal IGFBP3-expressing hDPSCs (denoted as lv5 hDPSCS). Odontogenic and osteogenic markers were compared between IGFBP3-over hDPSCs and lv5 hDPSCS at three time points: before osteogenic induction (0 d), and 4 days (4 d) and 7 days (7 d) afterwards. At each time point, compared with lv5 hDPSCS, IGFBP3-over hDPSCs had a lower level of odontogenic (DSPP and DMP1) and osteogenic (OSX, OPN, ALP and OCN) markers (Fig. 3A b–d). This trend proved more prominent after osteogenic induction. The IGFBP3 expression response to osteogenic induction was investigated using hDPSCs. RT-qPCR showed that the expression of IGFBP3 gradually decreased as mineralization process progressed (Fig. 3B). RT-qPCR and immunofluorescence analysis also showed IGFBP3 overexpression decreased OPG/RANKL ratio, which is a benchmark for mineralization microenvironment (Additional file 1: Fig. S1). In summary, IGFBP3 over-expression is associated with odontogenic and osteogenic marker suppression, and with osteogenic induction, the decreased IGFBP3 level in lv5-hDPSCs and greater differences in IGFBP3 between two groups of hDPSCs is associated with greater gaps in odontogenic and osteogenic markers between these two groups. These results demonstrate an inverse correlation between both odontogenic and osteogenic markers versus IGFBP3 expression. Western blot analysis further confirmed the effect of IGFBP3 over-expression (Fig. 3C).

Alizarin red and alkaline phosphatase staining was conducted to further estimate the difference in the osteogenic capability between lv5 hDPSCs and IGFBP3-over hDPSCs. Alkaline phosphatase staining showed a lower level of ALP in IGFBP3-over hDPSCs after osteogenic induction, compared with lv5 hDPSCS (Fig. 3D a, b). Alizarin red staining showed that mineral deposition of IGFBP3-over hDPSCs was also less prominent comparing with that of lv5 hDPSCs (Fig. 3D c, d).

Dental mesenchymal tissue from the P1 tooth germ was transfected with lentivirus to manipulate IGFBP3 expression. The transfection efficacy was evaluated with a fluorescence assay, where the signal strength in IGFBP3-over dental mesenchyme was found to be 1.89 times that of lv5 dental mesenchyme. RT-qPCR results revealed a significant decrease in the expression of Dspp and Ocn in samples of the dental mesenchyme with overexpression of IGFBP3 (Fig. 3E), which is consistent with the results of cell experiments.

### IGFBP3 inhibits osteogenic differentiation via Wnt/beta-catenin signaling.

**Fig. 4:**
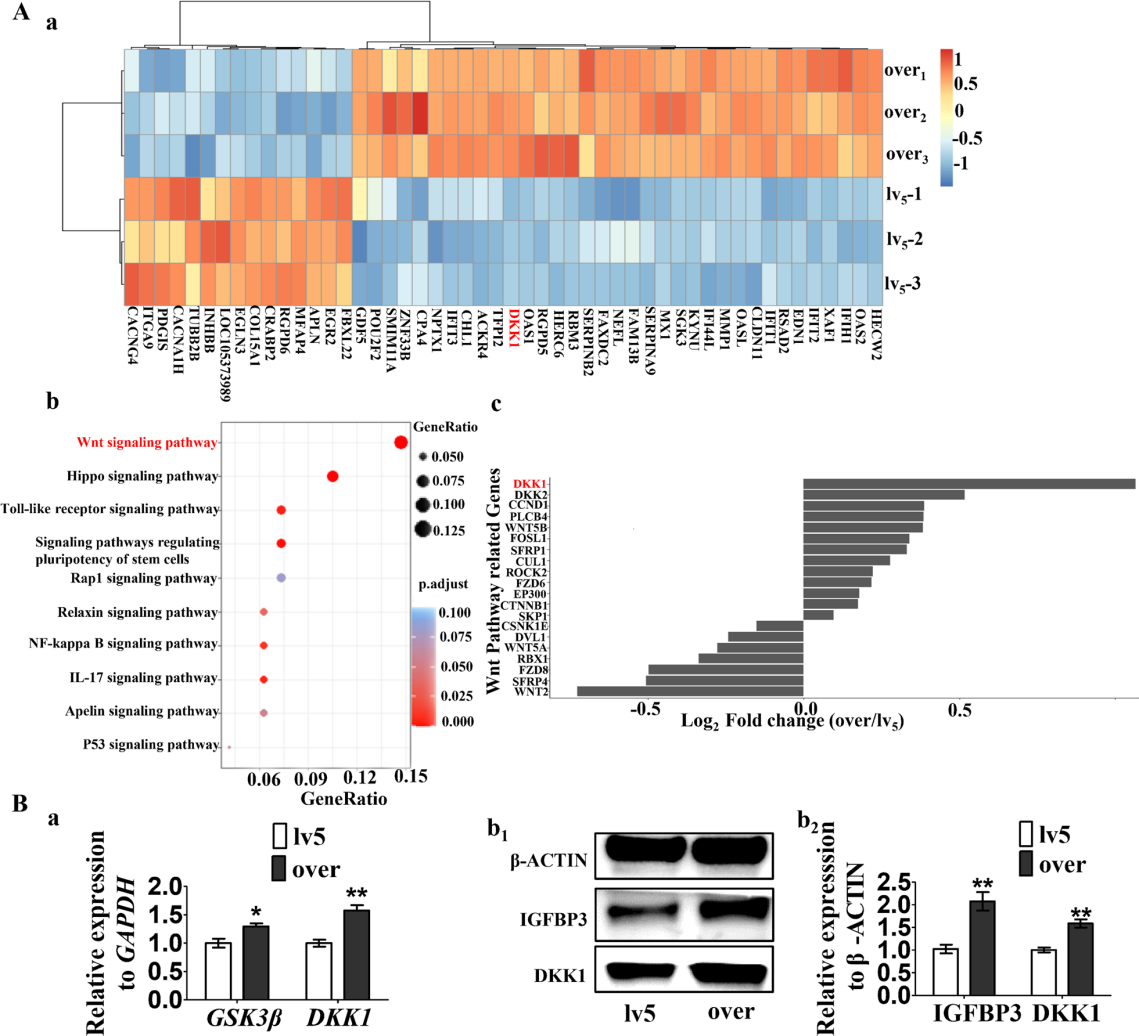
Bioinformatics analysis identifying differentially expressed genes (DEGs) between IGFBP3-over and lv5 hDPSCs. **A** DEGs between IGFBP3-over and lv5 hDPSCs were identified by RNA-seq analysis (a). KEGG pathway enrichment analysis revealed Wnt signaling pathway exhibited the highest enrichment score (b). The subsets of Wnt signaling pathway related genes in RNA-seq data were compared in terms of their expression levels and at the top of the list is Dickkopf related protein 1 (c). **B** Relative expression of *GSK3β* and *DKK1* in lv5 and IGFBP3-over hDPSCs were detected by RT-qPCR (a). The protein levels of IGFBP3 and DKK1 in lv5 and IGFBP3-over hDPSCs were detected by Western blot analysis (b1), with quantitative analysis of the relative protein expression (b2)

RNA-seq analysis was used to identify differential expressed genes between IGFBP3-over and lv5 hDPSCs (Fig. 4A a). With KEGG pathway enrichment analysis, several signaling pathways were identified as candidates for further research, among which the Wnt signaling pathway exhibited the highest enrichment score (Fig. 4A b). The subsets of Wnt signaling pathway related genes in RNA-seq data were then compared in terms of their expression levels. At the top of the list is Dickkopf-related protein 1 (DKK1), an inhibitor of the Wnt signaling pathway, whose expression level in IGFBP3-over hDPSCs was nearly twice as that in lv5 hDPSCs (Fig. 4A c). RT-qPCR analysis compared DKK1 and GSK3β levels between IGFBP3-over hDPSCs and lv5 hDPSCs, showing that IGFBP3 over-expression leads to an increased expression of both genes (Fig. 4B a). The western blot analysis also confirmed that the DKK1 protein level is increased in IGFBP3-over hDPSCs (Fig. 4B b1, b2)).

**Fig. 5:**
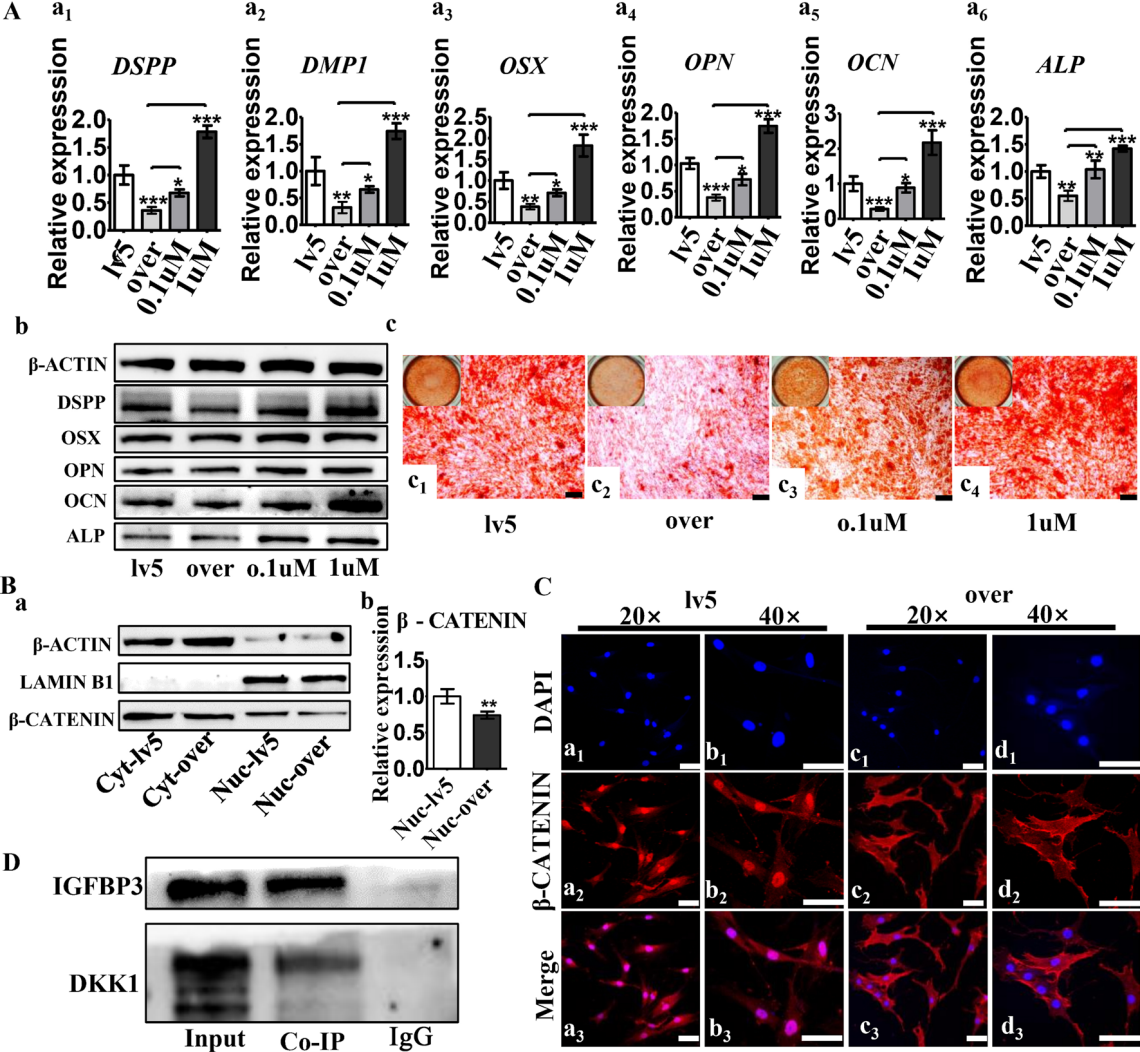
Results demonstrating IGFBP3 regulate Wnt signaling pathway via DKK1. **A** RT-qPCR compared tooth development related markers, including *DSPP* (a1) and *DMP1* (a2), and osteogenic markers, including *OSX* (a3), *OPN* (a4), *OCN* (a5) and *ALP* (a6) between lv5 hDPSCs, IGFBP3-over hDPSCs and IGFBP3-over hDPSCs treated with DKK1 inhibitor WAY-262611 (0.1 μM and 1 μM) for 14 days. Western blot analysis compared protein levels of DSPP, OSX, OPN, OCN and ALP between these groups (b). Alizarin red staining compared mineralized deposition between these groups after 14 days osteogenic induction, scale bar: 200 μm (c). **B** Location specific western blot analysis compared cytoplasmic beta-catenin (CTNNB1) levels and nuclear CTNNB1 levels between lv5 hDPSCs and IGFBP3-over hDPSCs (a), with quantitative analysis of nuclear CTNNB1 (b). Cyt: cytoplasmic, Nuc: nuclear, LAMIN B1 serve as the loading control for nuclear protein, β-ACTIN serve as the loading control for total protein. **C** Immunofluorescence analysis comparing CTNNB1 transnucleation by immunofluorescence. Scale bar: 50 μm. **(E)** Coimmunoprecipitation showed that anti-IGFBP3 pulled down DKK1 protein. n = 3, *p < 0.05, **p < 0.01, ***p < 0.001

The DKK1 inhibitor WAY-262611 was used to determine whether it can salvage the suppression of osteogenic and odontogenic markers expression caused by IGFBP3 over-expression. RT-qPCR and western blot analysis results demonstrate the DKK1 inhibitor reverse-elevated IGFBP3 influence on odontogenic (DSPP and DMP1) and osteogenic (OSX, OPN, OCN and ALP) markers in a dose-dependent manner (Fig. 5A a, b). These results were further supported by alizarin red staining (Fig. 5A c). The DKK1 inhibitor counteracts the inhibition of the matrix mineralization-inducing effect in hDPSCs caused by IGFBP3 over-expression.

Beta-Catenin intra-nuclear translocation is an essential step in the canonical Wnt signaling pathway. Western blot analysis of nuclear and cytoplasmic beta-catenin (CTNNB1) showed that compared with lv5 hDPSCs, IGFBP3-over hDPSCs had a lower level of intra-nuclear beta-catenin, while the cytoplasmic beta-catenin level was similar in two groups (Fig. 5B). Immunofluorescence analysis demonstrated a decrease in the overlap between DAPI and β-catenin in IGFBP3-over hDPSCs (Fig. 5C), further confirming the western blot analysis result. Co-Immunoprecipitation showed that IGFBP3 could pull down DKK1, suggesting that DKK1 is an integral part of the mechanism by which IGFBP3 inhibits Wnt/beta-catenin signaling. (Fig. 5D).

### Verification of the inhibition of osteogenic differentiation of IGFBP3-over hDPSCs in vivo.

**Fig. 6:**
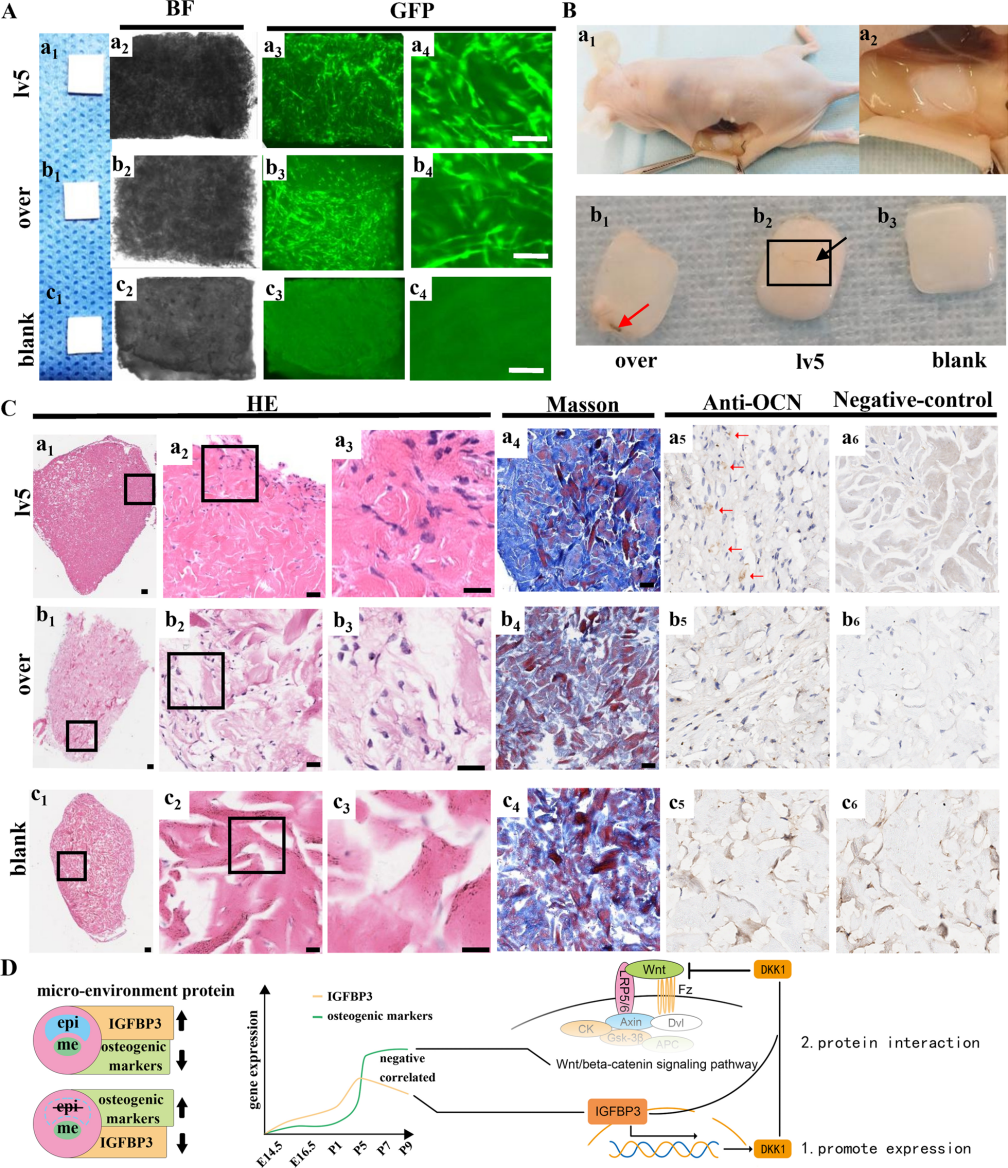
Verification of the inhibition of osteogenic differentiation of IGFBP3-over hDPSCs in vivo. **A** The hDPSCs and collagen membrane scaffold complex under the inverted phase contrast fluorescence microscope. BF channel: a_2_–c_2_; GFP channel: a_3_–c_3_, a_4_–c_4_. scale bar: 200 μm. **B** The hDPSCs-collagen complex removed from subcutaneous after culturing for 8 weeks in vivo. The red arrow indicates the degradation of collagen scaffold in the IGFBP3-over group (b_1_). The black frame and arrow indicate vascularization of the hDPSCs-collagen complex in the lv5 group (b_2_). **C** HE staining of tissue sections demonstrated massive degradation of the collagen membrane was found in the IGFBP3-over hDPSCs group (b_1_–b_3_), and there was no evident breakage of collagen fibers in lv5 hDPSCs group (a_1_–a_3_). Masson’s trichrome staining revealed less collagen fibers were stained (blue) in IGFBP3-over hDPSCs group comparing to lv5 hDPSCs group (a_4_–c_4_). Immunohistology assay results showing intense staining of OCN expression in lv5 group. The red arrows indicate parts of the tissue that were positive for immunohistochemical staining. scale bar: 250 μm, over: IGFBP3-over hDPSCs seeded in the membrane scaffold; lv5: lv5 hDPSCs seeded in the membrane scaffold; blank: no cell seeded in the membrane scaffold. **D** Schematic diagram summarizing the main findings of the present study

To further confirm these in-vitro findings and to study the effect of IGFBP3 overexpression on hDPSCs’ osteogenic differentiation over a long period of time, the hDPSCs-collagen complex was implanted into the subcutaneous space of nude mice. Before implantation, the hDPSCs and collagen membrane scaffold complex was observed under the inverted phase contrast fluorescence microscope. Both IGFBP3-over hDPSCs group and lv5 group exhibited strong green fluorescence signals, indicating successful formation of hDPSCs-collagen complex in both groups (Fig. 6A a1–b4). The blank collagen scaffold group yielded no cell signals (Fig. 6A c1–c4).

The hDPSCs-collagen complex was removed 8 weeks after implantation from the subcutaneous space. Compared with the lv5 group, the hDPSCs-collagen complex in the IGFBP3-over group was thinner, suggesting degradation of the scaffold. While vascularization of the hDPSCs-collagen complex was observed in the lv5 DPSCs group, there was no clear sign of it in the IGFBP3-over group (Fig. 6B). Novel bone-like tissue fabricated by hDPSCs was observed in both the IGFBP3-over and lv5 group, while no tissue was found in the collagen scaffold from the blank group. There was no evident breakage of collagen fibers in lv5 hDPSCs group, whereas massive degradation of the collagen membrane was found in the IGFBP3-over hDPSCs group (Fig. 6C a1–c3). Masson’s trichrome staining further confirmed this finding (Fig. 6C a4–c4). Immunohistochemical staining demonstrated a high level of OCN in the hDPSCs-collagen complex in the lv5 group, and relatively low levels of OCN in the IGFBP3-over group (Fig. 6C a5–c6). These results confirm that IGFBP3 has the ability to inhibit osteogenic differentiation in vivo.

## Discussion

Tooth loss and defect in tooth structure are common dental disease. Currently, oral restoration treatment is the mainstream approach. But tooth regenerative therapy are taking a more prominent position and it is the main focus of dental research [9]. A throughout understanding of tooth development process is the foundation for it. During tooth development, epithelial and mesenchymal tissues interact with each other in sequential and reciprocal manners, which is crucial for the process [10-12]. Molecular regulators secreted in the autocrine or paracrine manner constitute an important messenger system for epithelial mesenchymal interaction. In our previous study [3], protein micro-array analysis revealed that the removal of dental epithelium changed the expression of various secreted proteins. Many of them are involved in regulation of mineralization micro-environment.

Studies have shown that decellularized dentin matrix or bone matrix promotes biomineralization and odontogenesis by providing a mineralization micro-environment. Consistent with these results, we found osteogenic markers in tooth germ tissue increased during postnatal period. Bone morphogenetic proteins (BMPs) have been suggested to play a role in initiation and regulation of mineralization micro-environment [13, 14]. As a member of the BMPs family, BMP2’s promoting effect on osteogenic differentiation in various types of stem cells and hard tissue formation were validated by both in vitro and in vivo studies [15, 16]. Our result showed that BMP2 treatment further promotes expression of the osteogenic biomarkers (Runx2, Osx, Alp) and odontogenic biomarkers (Dspp, Dmp1, Ambn) in tooth germ extracted from post-natal mice (P7). Interestingly, we found BMP2 induced mineralization micro-environment played a different role during pre-natal period of tooth development. Consistent with previous study [11], osteogenic markers expression was kept at low level, and didn’t response to BMP2 treatment, indicating that osteogenic differentiation of mesenchymal stem cells during early phase of tooth development is negatively regulated. Furthermore, BMP2 treatment disrupted normal tooth development, which is manifested as odontogenic biomarkers expression suppression and dentin malformation. The contrast between BMP2’s action in E14.5 tooth germ and P7 tooth germ can be related to its expression pattern during tooth development. At the cap stage BMP2 is mainly localized inside epithelial region, but in postnatal days, it is continually expressed in dental papilla and adjacent tissues including cementoenamel junction and hertwig’s epithelial root sheath [17]. Together with our prior finding [3], a dual action for mineralization micro-environment in tooth development can be concluded: disruptive to odontogenesis process during early phase and promotive to osteogenesis/odontogenesis at later stage.

By analyzing data from our previous study [3], we found that after dental epithelium removal, in contrast to elevated osteogenic markers, insulin-like growth factor binding protein-3 (IGFBP-3) level decreased significantly. We validated this finding with RT-qPCR, and further found out that Igfbp3 is predominantly expressed in the mesenchymal region of tooth germ under the stimulation of epithelium. On temporal expression pattern of Igfbp3, we found that it increased gradually from the early stage of tooth development and peaked at P5, also, a negative correlation between Igfbp3 and osteogenic markers, especially during later stages of tooth development was found. Previous studies have shown that IGFBP3 suppresses osteogenic differentiation via binding with BMP2 [4, 18]; however, for dental papilla stem cells its role remained unclear. Our results show that IGFBP3 up-regulation inhibits osteogenic and odontogenic markers in both hDPSCs and dental mesenchymal tissue. We further performed in vivo experiments demonstrating that long-term IGFBP3 over-expression inhibits osteogenic differentiation of hDPSCs and causes massive degradation of the collagen scaffold.

One of the main roles for IGFBP3 is regulation of IGF-1 turn-over. IGF-1 can then antagonizes the Wnt/β-Catenin pathway [19]. But our RT-qPCR result (Additional file 2: Fig. S2) showed that IGFBP3 overexpression in hDPSCs led to decrease in IGF-1 expression, suggesting an IGF-1 independent pathway for its regulation on mineralization microenvironment. To investigate the underlying mechanism for IGFBP3’s influence on osteogenic/odontogenic differentiation of hDPSCs, we performed an RNA-seq study and pathway enrichment analysis. Results show that the genes related to Wnt/beta-catenin signaling pathway which plays an important part in osteogenic/odontogenic differentiation regulation [20-23], are actively modulated by IGFBP3. This finding is further supported by immunofluorescence analysis and the western blot result, which shows increased intra-nuclear translocation of beta-catenin. Among molecules related to Wnt/beta-catenin signaling pathway, IGFBP3 overexpression increase expression of DKK1 most significantly. Furthermore, the DKK1 inhibitor WAY-262611 could reverse the IGFBP3’s influence on osteogenic and odontogenic markers in a dose-dependent manner. These results indicate that DKK1 is one of key components for IGFBP3 to modulate the Wnt/beta-catenin signaling pathway.

Previous studies have shown that IGFBP3 and DKK1 both were capable of blocking the Wnt/beta-catenin signaling pathway [24], with different underlying mechanisms for these two molecules [25, 26]. In the current study, we demonstrated that IGFBP3 could bind with DKK1. The direct interaction between IGFBP3 and DKK1 would lead to their spatial aggregation, promoting synergistic suppression on the Wnt/beta-catenin signaling pathway. This could provide novel insight into the network of regulating mechanisms of the mineralization micro-environment during tooth development, and the functions of the IGFBP3-DKK1 complex are worth further exploration.

## Conclusions

In conclusion, we showed for tooth development, mineralization micro-environment is detrimental during early stage and beneficial in later stage. IGFBP3, a secreted protein mainly produced in the dental mesenchyme region under the stimulation of dental epithelium, was shown to inhibit the mineralization micro-environment, and its expression pattern is regulated in accordance with the needs of the mineralization microenvironment during tooth development. IGFBP3 plays the role of an escort to ensure the normal development of teeth, via directly interaction with DKK1, suppressing the Wnt/beta-catenin signaling pathway, and negatively regulate the osteogenic/odontogenic differentiation of dental papilla stem cells. Our data provide novel insight into IGFBP3’s role in regulation of mineralization microenvironment and enhanced our knowledge about tooth regenerative therapy.

## Methods

### Animal study and tissue isolation

We isolated specimens of tooth germs at different stages of tooth development for in vitro experiments. The ‘tooth germ’ is an aggregation of cells that eventually forms a tooth. To obtain mouse embryos and tooth germs for histological evaluation, 17 pregnant Chinese Kunming (KM) mice (4 weeks old), 18 Chinese Kunming mice (1, 3, 5, 7, and 9 days old) were purchased from San Yet-sun University. 9 Male nude mice (5 weeks old) were purchased for in vivo experiments. For experiments involving the isolation of tooth germs and the mesenchyme, three mice were used for each group, originating from the same pregnant mouse to minimize genetic differences. The study protocol for animal studies was approved by the ethics committee of the Hospital of Stomatology, Sun Yat-sen University (ERC-2013-15; Guangzhou, China).

To study the influence of spatial and temporal expression patterns of IGFBP3 along with various markers, as well as influence of osteogenic induction on tooth development in a controlled environment, organ isolation was performed. One Pregnant KM mouse was sacrificed to obtain three mouse embryos at each time point (embryonic day 14.5 (E14.5) and 16.5 (E16.5)), and three KM mice were sacrificed at each time point (postnatal day 1 (P1), 3(P3), 5(P5), 7(P7) and 9 (P9)), whose mandibles were isolated surgically.

The first mandibular molar tooth germs were dissected from the E14.5 and E16.5 KM mouse embryo, and P1, P3, P5, P7, and P9 KM mice. For the tooth germs of E14.5 and P1, epithelium mesenchyme dissociation was performed according to the protocol specified in our previous study [3]. Tooth germs and the dental mesenchyme were cultured on six-well transwell chambers with 8 µm pores (Falcon Corning, Corning, NC, USA) at 37 ºC in 5% CO_2_ with 1 mL/well of Dulbecco's modified eagle's medium (DMEM, Gibco, Waltham, MA, USA) supplemented with 10% fetal bovine serum (FBS, Gibco), 100 µg/mL of ascorbic acid (Sigma, St. Louis, MO, USA), and 2 mM of L-glutamine (Gibco). Recombined mouse bone morphogenetic protein 2 protein (BMP2, 200 ng/mL; 120–02; Pepro Tech, NJ, USA) was added to the culture medium of the E14.5 and P7 tooth germ to study the effect of osteogenic inducing micro-environment on tooth development. The lentivirus method was employed to obtain the dental mesenchyme overexpressing Insulin-like growth factor binding protein 3 (Igfbp3). The culture medium was changed every 3 days.

### Cell isolation and culture

Freshly extracted healthy third molars without caries were collected from patients (10–16 years of age) at the Hospital of Stomatology, Sun Yat-sen University after obtaining informed written consent. The procedure conformed to the guidelines and protocols of the Ethical Committee in Hospital of Stomatology, Sun Yat-sen University. The tooth germs of the third molars were still embedded in the alveolar bone at this time, they were pulled out carefully to avoid damaging the dental papilla under the hard tissue. Subsequently the dental papilla tissue was immersed in alpha modified Eagle medium quickly and aseptically processed with 2% penicillin–streptomycin solution after the tooth germs extraction. HDPSCs were isolated from dental papilla tissue that digested with 3 mg/mL collagenase I (cat. no. 17100017; Gibco) for 30 min at 37 °C. Isolated hDPSCs were cultured with basal media, which contains alpha modified Eagle medium (α-MEM, Gibco), supplemented with 10% FBS, 100 U/mL penicillin and 100 mg/mL of streptomycin (Sigma), then passaged before reaching 80% confluence, then seeded at a density of 10^5^ cells/mL. Three days after seeding, to stimulate osteogenic differentiation, hDPSCs were treated with mineralization induction medium, which was prepared according to the guidelines of a reported study [27] with some modifications—the basal media was supplemented with 10 nM dexamethasone (Sigma), 50 μg/mL L-ascorbic acid (Sigma) and 10 mM β-glycerophosphate disodium (Sigma). HDPSCs at passages 3–5 were used for experiments and cultured at 37 °C in a 5% CO_2_ humidified incubator. The culture media were changed every 3 days.

### IGFBP3 expression manipulation

Full-length IGFBP3 (Igfbp3) cDNA was cloned from total RNA and inserted into a pGLV5 vector to obtain overexpressing lentivirus particles. Lentivirus particles containing empty pGLV5 plasmid were purchased from HANBIO (HANBIO). HDPSCs and the dental mesenchyme were transfected with lentivirus particles containing IGFBP3 (Igfbp3)-overexpressing pGLV5 or empty pGLV5 vector for 16 h in the presence of 5 µg/mL polybrene. The fluorescence microscope confirmed that the dental mesenchyme and > 80% of transfected cells positively expressed the green fluorescence protein. The transfection efficiency was further confirmed by real-time quantitative reverse transcription polymerase chain reaction (RT-qPCR) and western blot analysis, which proved the successful transfection of IGFBP3 into hDPSCs and dental mesenchyme.

### Histology evaluation

#### Immunohistochemical staining

Immunohistochemistry analysis was performed on mandibles tissue dissected from P1, P3, P5, P7, and P9 KM mice to evaluate IGFBP3 expression during tooth development, and also on first mandibular tooth germs from E14.5 KM mouse embryo and P7 KM mice, which were treated with recombinant BMP2 protein to evaluate the effects of a high mineralization micro-environment on tooth development. Immunohistochemistry staining was also performed on the hDPSCs-collagen complex obtained from in vivo experiments to evaluate the long-term influence of IGFBP3 on hDPSCs. Mandibles of P1–P9 were resected, then fixed in 4% paraformaldehyde for 72 h at room temperature. Then, they were transferred to ethylenediamine tetra acetic acid for decalcification for 2 weeks. The hDPSCs-collagen complex obtained from nude mouse subcutaneous tissue was fixed in 4% paraformaldehyde at room temperature for 72 h without decalcification.

Fixed tissues were dehydrated with graded alcohol and embedded in paraffin, and subsequently cut into 4-μm-thick sections. The sections were dewaxed, rehydrated, and soaked in a citrate buffer (pH 8.0) at 100 °C for 10 min in an autoclave for heat-mediated antigen repair. To reduce non-specific background staining caused by endogenous peroxidase, sections were incubated in a hydrogen peroxide solution to block for 15 min at room temperature. Then sections were incubated with 10% normal goat serum for 20 min to block nonspecific antibody binding. Next, the anti-IGFBP3 polyclonal antibody (1:500, ab217205, Abcam, Cambridge, UK) was used as the primary antibody, and sections were incubated overnight in a humid chamber at 4 °C. Meanwhile, the negative control was incubated with PBS instead. After washing with PBS, sections were incubated with peroxidase-labeled secondary antibodies for 1 h at room temperature. Afterward, a diaminobenzidine (DAB) detection kit (Polymer) (Gene Tech, Shanghai, China) was used. DAB plus chromogen were added dropwise into the DAB plus substrate, mixed, and dropped onto the sections to incubate for 10 min. Hematoxylin was used for nuclear counterstaining. Finally, the stained slides were dehydrated and mounted. Slides were photographed to observe IGFBP3 expression in the mandibles tissue from P1 to P9. Hematoxylin–eosin (HE) staining and Masson's trichrome staining were used to evaluate the tooth structure after stimulation with 200 ng/mL of BMP2.

#### Immunofluorescence analyses

Immunofluorescence analyses were conducted to evaluate the nuclear translocation of β-catenin. HDPSCs were seeded on a confocal dish at a density of 10^3^ cells/well and cultured in the mineralization induction medium for 4 days. Cells were fixed with 4% PFA for 20 min and permeabilized with 0.3% Triton X-100 in PBS for 10 min, after which they were incubated overnight with the anti-β-catenin polyclonal antibody (1:800; 8814; Cell Signaling Technology, Danvers, MA, USA) at 4 °C. The following day, after washing with PBS, cells were incubated with fluorescein-conjugated goat anti-rabbit antibody (1:200, DyLight 594, A23420; Abbkine, CA, USA) for 1 h in a dark chamber. Subsequently, the cells were counterstained with 4′6-diamidino-2-phenylindole (DAPI; Beyotime, Shanghai, China) for 10 min for nuclear labeling. Finally, we captured images using a confocal laser scanning microscope (Olympus, Tokyo, Japan).

### Osteogenic differentiation

#### Alizarin red staining

HDPSCs were seeded into 12-well plates at a density of 1.0 × 10^5^ cells per well and cultured in the mineralization induction medium for 14 days. Alizarin red staining (ARS) was used to observe the mineral deposition between the lv5 hDPSCs and IGFBP3-over hDPSCs after osteogenic induction. The cells were first fixed with 10% neutral-buffered formalin solution for 30 min, and stained with 1% Alizarin Red-S solution (Sigma) for 5 min at room temperature. The cells were carefully rinsed with deionized water and allowed to dry. Red staining indicated the formation of mineral nodules, which were observed and photographed by a phase contrast microscope.

#### Alkaline phosphatase staining

The seeding of hDPSCs was carried out into six-well plates, followed by incubating with the mineralization induction medium for 7 days. Differences in the osteogenic capability between blank and transfected hDPSCs were compared by alkaline phosphatase staining. The culture medium was aspirated, and the cells were washed with 500 μL Tris-buffered saline, 0.05% Tween 20 (TBST). Then, 1 mL of 4% paraformaldehyde was added and incubated at room temperature for 5 min. The cells’ alkaline phosphatase (ALP) activity was detected by a TRAP/ALP stain kit (Wako, Tokyo, Japan) according to the manufacturer recommendations. Images were acquired using the same camera parameters.

### Quantitative reverse transcription-polymerase chain reaction (RT-qPCR)

Total tissue RNA was extracted from tooth germs or isolated dental mesenchyme using Purelink® RNA Mini Kit (Invitrogen, Waltham, MA, USA) according to the manufacturer’s instructions. Total cell RNA was extracted using the RNA-quick Purification Kit (Yishan, Shanghai, China) and the RNA was reversed transcribed using PrimeScript™ RT reagent (Takara, Tokyo, Japan). Subsequently, messenger RNA expression was measured by RT-qPCR using qPCR SYBR Green detection reagent (Yeasen, Shanghai, China). Data were analyzed by formula 2^−∆∆CT^ with glyceraldehyde-3-phosphate dehydrogenase (GAPDH) as an internal reference to normalize RT-qPCR results. The sequences of the mRNA primers are listed in Additional file 3: Table S1.

### Western blot analysis

IGFBP3-over hDPSCs, lv5 hDPSCs were seeded at a density of 1 × 10^6^ cells per well. Total proteins were extracted using RIPA buffer according the manufacturer’s protocol. Cell samples in six-well plates were washed with PBS and lysed in RIPA lysis buffer (Cwbio, Beijing, China) with a 1% protease inhibitor cocktail (Cwbio) on ice for 30 min. Lysates were centrifuged at 14,000×*g* for 15 min, and supernatants were collected. Nuclear protein was extracted with a nuclear protein extraction kit following to the manufacturer’s instructions (Thermo Fisher Scientific, Waltham, MA, USA). We used the BCA protein assay kit (Cwbio) to detect protein concentration and then added SDS-PAGE loading buffer (Cwbio) to the lysates. Mixtures were boiled at 99 °C for 10 min. We separated the same amount of protein with 4–12% SDS-PAGE gel (GenScript, Piscataway, NJ, USA) and transferred the protein in the gels to PVDF membranes (Millipore Corp, Billerica, MA, USA). Membranes were blocked with 5% skimmed milk in TBST for 1 h and incubated overnight with primary antibodies at 4 °C. The primary antibodies were diluted as follows: anti-IGFBP3 (1:1000; 25,864 Cell Signaling Technology), anti-DSPP (1:500, bs-8557R; Bioss, Beijing, China), anti-OSX (1:1000; DF7731; Affinity Biosciences, Cincinnati, OH, USA), anti-OPN (1:200; sc-21742; Santa Cruz Biotechnology, Santa Cruz), anti-OCN (1: 200; sc-365797; Santa Cruz Biotechnology), anti-ALP (1:500; ab229126; Abcam), anti-β-catenin (1:1000; 8814; Cell Signaling Technology) and anti-β-actin (1:1000; GB11001; Servicebio, Wuhan, China) as controls for total proteins, anti-laminB1 (1:500; GB111802; Servicebio) as controls for the nuclear proteins. The following day, after washing with TBST, the membranes were incubated with HRP-conjugated anti-rabbit secondary antibody (1:3000; GB23303/GB23301; Servicebio) for 1 h at room temperature, and visualized with the chemiluminescent HRP substrate (Millipore Corp). All assays were performed in triplicate. Image J software, version 1.5 [28] (National Institutes of Health, Bethesda, MD, USA) was used to quantify protein expression levels.

### Coimmunoprecipitation

Coimmunoprecipitation was performed to investigate the potential mechanism of the interaction between IGFBP3 and DKK1. Total proteins were extracted from IGFBP3-over hDPSCs 72 h post-transfection. A small amount of lysate was used as a positive control for western blot analysis (Input), the remaining lysates were divided into two groups as Co-IP and IgG. They were incubated respectively with anti-mouse IGFBP3 (2 ug; sc-374365; Santa Cruz Biotechnology) and anti-mouse IgG (2 ug; GB111739; Servicebio) at 4 °C overnight. Protein A/G magnetic beads (HY-K0202, MedChemExpress, Monmouth Junction, NJ, USA) were washed with binding buffer (0.5% PBST), after which they were added to the cell lysate incubated with the antibody and incubated at 4 °C for 2 h to couple the antibody with Protein A/G magnetic beads. Magnetic separation was performed to collect magnetic beads. Subsequently, the SDS-PAGE loading buffer was added to the magnetic beads, and the mixtures were boiled at 99 °C for 10 min, separating the magnetic beads and collecting the supernatants for SDS-PAGE analysis. The primary antibody was anti-rabbit IGFBP3 (1:1000; 25,864; Cell Signaling Technology), and anti-rabbit DKK1 (1:1000; ab109416; Abcam). To avoid the influence of light and heavy chains of IgG on the target bands, the HRP-conjugated anti-rabbit secondary antibody was selected, and the species was different from the antibody of Co-IP and IgG, which is a mouse monoclonal antibody.

### Protein microarrays

Protein microarray assays were carried out to compare the expression of various extracellular cytokines between tooth germs and those in isolated dental-mesenchyme tissue according to the previously reported protocol [3]. The fold-change in the protein level was calculated using the following equation: Fold-Change = log_2_(V_mesenchyme_/V_tooth germ_), where V_mesenchyme_ denotes the reading for that protein from the isolated dental‑mesenchyme culture fluid, and V_tooth germ_ is the microarray reading for expression of a certain protein from tooth-germ culture fluid. Significantly differential proteins were searched using *p* < 5e−5 which was adjusted with Bonferroni correction.

#### RNA-Seq

IGFBP3-over hDPSCs, lv5 hDPSCs were cultured for 7 days to carry out RNA-Seq. Total RNA was extracted using the Trizol reagent (Thermo Fisher) according to the manufacturer’s instructions. Then, RNA-Seq was conducted according to a previously reported protocol [29].The libraries were constructed by NEBNext Ultra for Illumina. RNA sequencing was performed using the Illumina HiSeq4000 sequencer. Raw reads were processed by removing adaptor reads and poor-quality tags. Clean reads were then used for subsequent analyses, and the obtained data were indicated as fragments per kilobase per million (FPKM) reads values and quantified by RSEM software. All DEGs were mapped to terms in the GO databases. Significantly enriched GO terms were searched for among the DEGs using *p* < 0.05 as the threshold, All DEGs were mapped to the KEGG database, and searched for significantly enriched KEGG pathways at the *p* < 0.05 level.

#### Subcutaneous transplantation of hDPSCs-collagen membrane scaffolds complex

Collagen membrane scaffolds were seeded with IGFBP3-over hDPSCs or lv5 hDPSCs to form the hDPSCs-collagen membrane scaffolds complex. The collagen membrane was cut into 4 × 4 mm pieces and placed in 24-well plates with α-MEM and fully immersed for 4 h, then dried in the incubator at 37 °C for 30 min before seeding the cells. In each well, 20 μL of cell suspension containing 10^5^ cells was seeded on the collagen membrane and incubated for 2 h at 37 °C. Then, 100 μL of basal media was added to the edge of each well of a 24 well plate and incubated for 2 h. Finally, 1 mL of basal media was added to the 24-well plates. After 24 h, the hDPSCs-collagen membrane scaffolds complex was observed under the inverted phase contrast fluorescence microscope, indicating large numbers of hDPSCs adhered to the collagen membrane scaffold. The constructed hDPSCs-collagen membrane scaffolds complex was cultured for 48 h with mineralization induction medium and implanted into subcutaneous tissue of nude mice. After 8 weeks, mice were sacrificed, and the degradation of the tissue engineered compound as well as osteogenic differentiation of hDPSCs were evaluated by HE, Masson, and IHC staining.

#### Statistical analysis

GraphPad Prism 8 (GraphPad, La Jolla, CA, USA) and SPSS 25.0 (IBM Corporation, Armonk, NY, USA) were used for statistical analysis. All data are presented as the mean ± standard error from at least three independent experiments. Upon confirmation of a normal distribution, all quantitative data were subjected to Student’s t tests (comparison between two groups) or one-way ANOVA (comparison among groups), *p* < 0.05 was considered statistically significant. Heatmaps for differential expression of proteins and gene were created with the pheatmap package (R 4.0, r Foundation).

## Supplementary Information

**Additional file 1**. Overexpression of IGFBP3 disturbs the OPG/RANKL axis. (A)RT-qPCR revealed the mRNA levels of OPG, RANKL and OPG/RANKL ratio,** p < 0.01, *** p < 0.001. (B) The expression and localization of OPG and RANKL proteins in lv5 and IGFBP3-over hDPSCs were compared by immunofluorescence analysis. 20×scale bar: 100μm, 40×scale bar: 50μm.

**Additional file 2**. Relative expression of IGFI and IGFIR in lv5 and IGFBP3-over hDPSCs were detected by RTqPCR.* p < 0.05.

**Additional file 3**. List of primers’ sequence in this study.

## Data Availability

The datasets used and analyzed during the current study are available from the corresponding author on reasonable request.

## Abbreviations

ALP: Alkaline phosphatase staining
ANOVA: Analysis of variance
ARS: Alizarin red staining
BCA: Bicinchoninic acid
BMP: Bone morphogenetic protein
cDNA: Complementary DNA
Co-IP: Coimmunoprecipitation
CTNNB1: Beta-catenin
DAB: Diaminobenzidine
DAPI: 4′,6-Diamidino-2-phenylindole
DEGs: Differentially expressed genes
DKK1: Dickkopf-related protein 1
DMEM: Dulbecco’s modified eagle’s medium
DMP1: Dentin matrix protein 1
DSPP: Dentin sialophosphoprotein
Eda: Ectodysplasin
EMI: Epithelial mesenchymal interactions
FBS: Fetal bovine serum
FGF: Fibroblast growth factor
FPKM: Fragments per kilobase per million
GAPDH: Glyceraldehyde-3-phosphate dehydrogenase
hDPSCs: Human dental papilla stem cells
HE: Hematoxylin-eosin
HRP: Horseradish peroxidase
IGF: Insulin-like growth factor
IGFBP3: Insulin-like growth factor binding protein 3
IgG: Immunoglobin G
IHC: Immuno-histochemistry
KEGG: Kyoto encyclopedia of genes and genomes
KM: Kunming
ME: Dental mesenchyme alone
OCN: Osteocalcin
OPN: Osteopontin
OSX: Osterix
PBS: Phosphate buffered saline
PBST: Phosphate buffered saline with Tween-20
PFA: Paraformaldehyde
PVDF: Polyvinylidene difluoride
RIPA: Radioimmunoprecipitation assay buffer
RNA-Seq: RNA sequencing
RT-qPCR: Quantitative reverse transcription PCR
SDS-PAGE: Sodium dodecyl sulfate polyacrylamide gel electrophoresis
Shh: Sonic Hedgehog
TBST: Tris-buffered saline, 0.05% Tween 20
TG: Whole tooth germ
TGF: Transforming growth factor
TG-ME: Dental mesenchyme isolated after culture
TRAP: Tartrate-resistant acid phosphatase
α-MEM: Alpha modified Eagle medium
