# Supplementary figures and images for: Dynamic expression of IGFBP3 modulate dual actions of mineralization micro-environment during tooth development via Wnt/beta-catenin signaling pathway
Source: Biol Direct. 2023 Jun 26;18:34. doi: 10.1186/s13062-023-00391-9 (PMC10291802; doi:10.1186/s13062-023-00391-9)

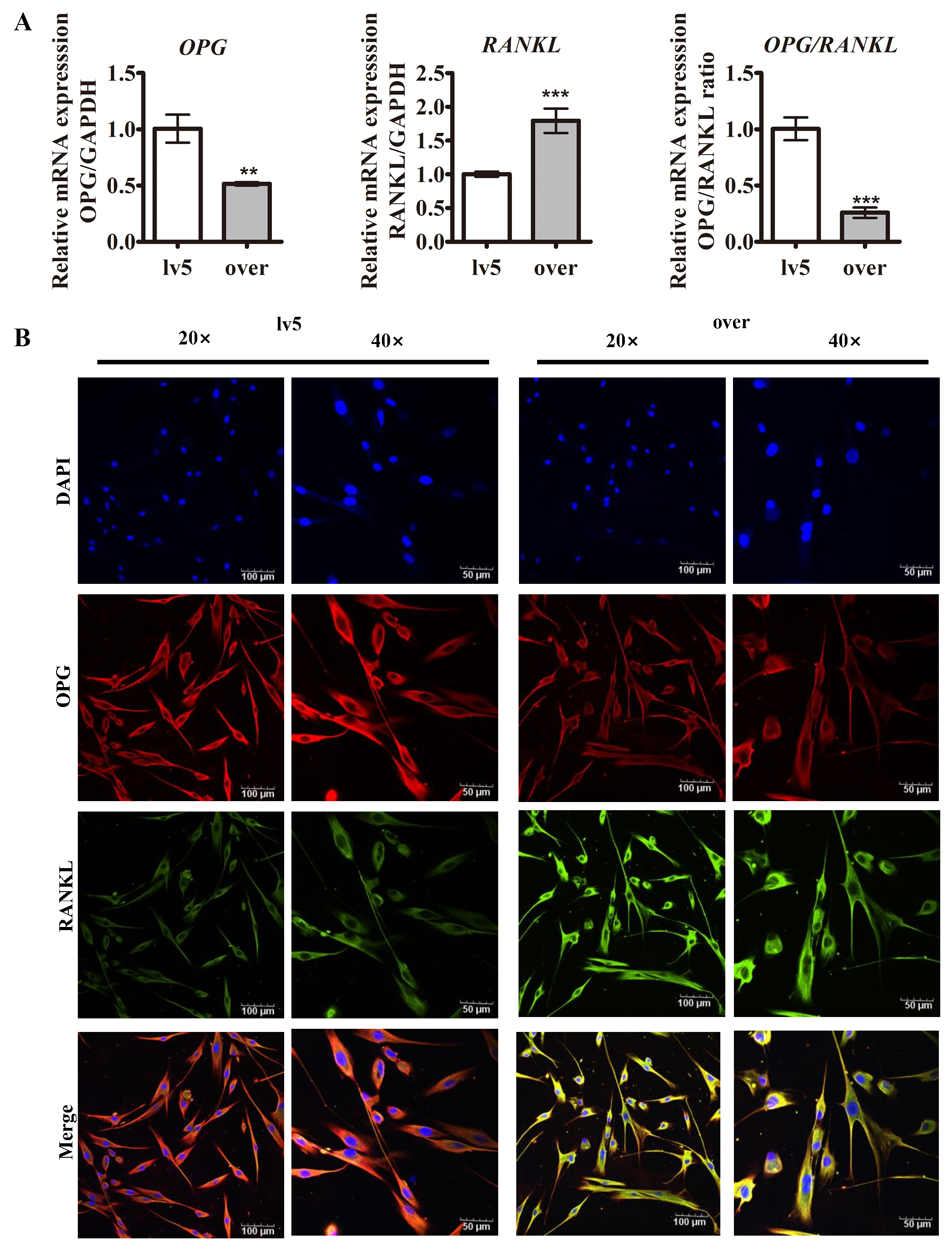

Supplement: Supplementary file 1 — Additional file 1. Overexpression of IGFBP3 disturbs the OPG/RANKL axis. (A)RT-qPCR revealed the mRNA levels of OPG, RANKL and OPG/RANKL ratio,** p < 0.01, *** p < 0.001. (B) The expression and localization of OPG and RANKL proteins in lv5 and IGFBP3-over hDPSCs were compared by immunofluorescence analysis. 20×scale bar: 100μm, 40×scale bar: 50μm. [file 13062_2023_391_MOESM1_ESM.jpg]

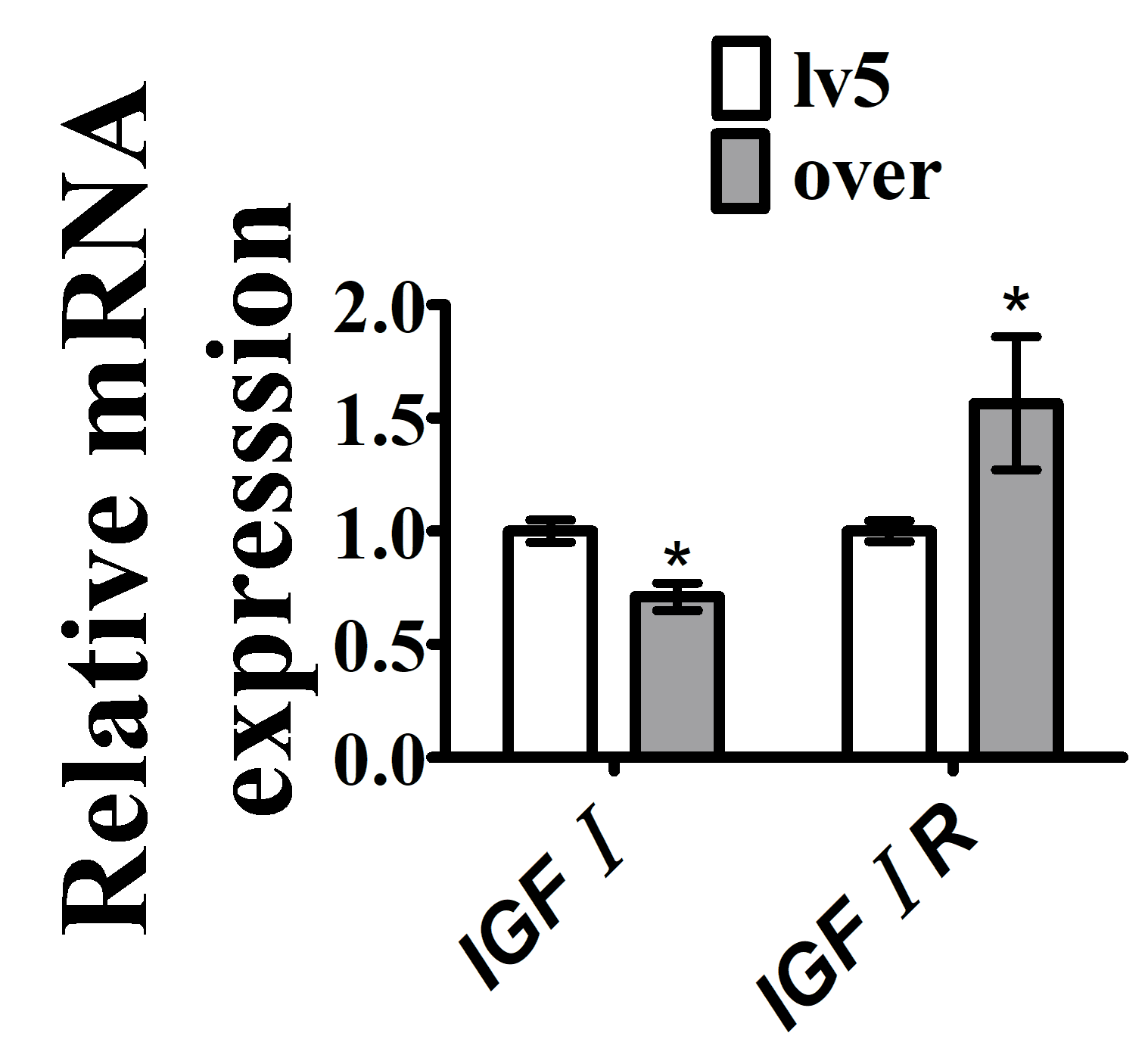

Supplement: Supplementary file 2 — Additional file 2. Relative expression of IGFI and IGFIR in lv5 and IGFBP3-over hDPSCs were detected by RTqPCR.* p < 0.05. [file 13062_2023_391_MOESM2_ESM.tif]
