# Supplementary material for: Dynamic expression of IGFBP3 modulate dual actions of mineralization micro-environment during tooth development via Wnt/beta-catenin signaling pathway
Source: Biol Direct. 2023 Jun 26;18:34. doi: 10.1186/s13062-023-00391-9 (PMC10291802; doi:10.1186/s13062-023-00391-9)
Supplement: Supplementary file 3 — Additional file 3. List of primers’ sequence in this study. [file 13062_2023_391_MOESM3_ESM.docx]

| species | gene | forward/reverse | primer(5' to 3') |
| --- | --- | --- | --- |
| Mus musculus Km | Gapdh | forward | AAGAAGGTGGTGAAGCAGGCATC |
|  |  | reverse | CGGCATCGAAGGTGGAAGAGTG |
|  | Igfbp3 | forward | CACACCGAGTGACCGATTCC |
|  |  | reverse | GTGTCTGTGCTTTGAGACTCAT |
|  | Bmp2 | forward | AACACCGTGCGCAGCTTCCATC |
|  |  | reverse | CGGAAGATCTGGAGTTCTGCAG |
|  | Dspp | forward | ATGGCAAGTGAGATAGGAGAGC |
|  |  | reverse | GTGTTCCCCTGTTCGTTTACTAA |
|  | Dmp1 | forward | CAGAGGGACAGGCAAATAGTGAC |
|  |  | reverse | CATCGCCAAAGGTATCATCTCC |
|  | Ambn | forward | TTGAGCCTTGAGACAATGAGAC |
|  |  | reverse | AAGTCCGTGCACCATAAACTAT |
|  | Osx | forward | GGAAAGGAGGCACAAAGAAGC |
|  |  | reverse | CCCCTTAGGCACTAGGAGC |
|  | Opn | forward | CGACGATGATGACGATGATGAT |
|  |  | reverse | CTGGCTTTGGAACTTGCTTGAC |
|  | Ocn | forward | CCGCCTACAAACGCATCTAT |
|  |  | reverse | GAGAGGACAGGGAGGATCAA |
|  | Alp | forward | CGGGACTGGTACTCGGATAA |
|  |  | reverse | ATTCCACGTCGGTTCTGTTC |
|  | Runx2 | forward | ACACTGCCACCTCTGACTTC |
|  |  | reverse | ATGAAATGCTTGGGAACTGCC |
| Homo sapiens | GAPDH | forward | CTGGGCTACACTGAGCACC |
|  |  | reverse | AAGTGGTCGTTGAGGGCAATG |
|  | IGFBP3 | forward | AGACACACTGAATCACCTGAAGT |
|  |  | reverse | AGGGCGACACTGCTTTTTCTT |
|  | Dspp | forward | GGAGCCACAAACAGAAGCA |
|  |  | reverse | TGGACAACAGCGACATCCT |
|  | Dmp1 | forward | AGGAAGTCTCGCATCTCAGAG |
|  |  | reverse | TGGAGTTGCTGTTTTCTGTAGAG |
|  | OSX | forward | TCCTCCTGCGACTGCCCTAA |
|  |  | reverse | GGTGCGAAGCCTTGCCATA |
|  | OPN | forward | ATGGAAAGCGAGGAGTTGA |
|  |  | reverse | GGCTGTCCCAATCAGAAGG |
|  | OCN | forward | CTCACACTCCTCGCCCTATT |
|  |  | reverse | CCTCCTGCTTGGACACAAA |
|  | ALP | forward | TAAGGACATCGCCTACCAGCTC |
|  |  | reverse | TCTTCCAGGTGTCAACGAGGT |
|  | DKK1 | forward | ATAGCACCTTGGATGGGTATTCC |
|  |  | reverse | CTGATGACCGGAGACAAACAG |
|  | GSK3β | forward | AGACGCTCCCTGTGATTTATGT |
|  |  | reverse | CCGATGGCAGATTCCAAAGG |
